# Maternal hemodynamics and computerized cardiotocography during labor with epidural analgesia

**DOI:** 10.1007/s00404-022-06658-2

**Published:** 2022-06-15

**Authors:** Stefano Raffaele Giannubilo, Mirco Amici, Simone Pizzi, Alessandro Simonini, Andrea Ciavattini

**Affiliations:** 1 Department of Obstetrics and Gynecology, Marche Polytechnic University, Ancona, Italy; 2 Department of Anaesthesia and Intensive Care, Salesi Hospital, Ancona, Italy; 3 Department of Clinical Sciences, Polytechnic University of Marche Salesi Hospital, via Corridoni 11, 60123 Ancona, Italy

**Keywords:** Analgesia, Cardiotocography, Labor, Hemodynamics

## Abstract

**Purpose:**

To analyze the mechanisms involved in the fetal heart rate (FHR) abnormalities after the epidural analgesia in labor.

**Methods:**

A prospective unblinded single-center observational study on 55 term singleton pregnant women with spontaneous labor. All women recruited underwent serial bedside measurements of the main hemodynamic parameters using a non-invasive ultrasound system (USCOM-1A). Total vascular resistances (TVR), heart rate (HR), stroke volume (SV), cardiac output (CO) and arterial blood pressure were measured before epidural administration (T0), after 5 min 5 (T1) from epidural bolus and at the end of the first stage of labor (T2). FHR was continuously recorded through computerized cardiotocography before and after the procedure.

**Results:**

The starting CO was significantly higher in a subgroup of women with low TVR than in women with high-TVR group. After the bolus of epidural analgesia in the low-TVR group there was a significant reduction in CO and then increased again at the end of the first stage, in the high-TVR group the CO increased insignificantly after the anesthesia bolus, while it increased significantly in the remaining part of the first stage of labor. On the other hand, CO was inversely correlated with the number of decelerations detected on cCTG in the 1 hour after the epidural bolus while the short-term variation was significantly lower in the group with high-TVR.

**Conclusion:**

Maternal hemodynamic status at the onset of labor can make a difference in fetal response to the administration of epidural analgesia.

## Introduction

Since the beginning of its history, analgesia in labor has always had controversial aspects and doubts about the maternal and fetus/neonatal consequences, thus safety remains a challenge to pursue. Transient abnormalities in fetal heart rate (FHR) have been described in up to 15% of the cases after the use of analgesia during labor [1] complicating the interpretation of fetal CTG and the prediction of a fetal acidemia at birth. Decelerations of FHR and bradycardia have been reported for all types of labor analgesia (epidural, spinal, combined spinal-epidural and intravenous opioids) [2]. The clinical significance of these changes is not entirely clear, however, there is a common consensus on maternal and fetal oxygenative and vascular pathophysiology [3]. It has been reported that the fetal oxygenation is altered with the dose dependent administration of epidural analgesia [4], as well has been proposed the hypothesis of uterine hyperactivity due to the reduction of catecholamines [5] or maternal hypotension due to an imbalance of adrenalin/noradrenalin ratio [6]. In this context several studies have described uteroplacental and fetal hemodynamics after labor analgesia with differences in clinical characteristics (antenatal, induction of labor, high risk or low risk pregnancies), vascular district evaluated, type of anesthesia (continuous infusion, single dose, self-controlled) and drugs used [7]. In the majority of studies, FHR changes is not associated with an increased incidence of cesarean section and did not appear to have an immediate effect on neonatal status as determined by Apgar scores [8]. Based on recent evidence of a maladaptive cardiovascular response to pregnancy complicated by placental syndromes [9–12], maternal hemodynamic assessment it has become an interesting way to evaluate maternal–fetal interactions from a different point of view. Labor and delivery are events that have a great impact on maternal general hemodynamics the change in maternal position from supine to lateral alone may produce an increase in cardiac output (+ 21.7%), decreased heart rate (− 5.6%), and increased maternal stroke volume (+ 26.5%) [13]. Anxiety, pain and exertion increases both heart rate and stroke volume, just as the utero-placental consequences of the reduced venous return to the heart due to caval compression from the supine position are well known. An increment in basal cardiac output of 12% has been reported in a group of women during labor [14].

The objective of the present study is to analyze the hemodynamic pattern of women during labor before and after epidural analgesia and its relationship with FHR.

## Patients and methods

This was a prospective unblinded single-center observational study carried out at Salesi Maternal-Neonatal University Hospital in Ancona (Italy), between March 2018 and June 2019. The center treats 1800 parturients per year, with an epidural analgesia rate in labor of 40% and a cesarean delivery rate of approximately 24%.

Fifty five low-risk pregnant women in active labor with normal FHR trace submitted to epidural analgesia were recruited. Inclusion criteria were: healthy single pregnancy after the 37th week of gestation, spontaneous active labor (cervical dilation of at least 3 cm), age 18–40 years, height 155–180 cm, body mass index < 35 kg/m^2^, normal FHR pattern at admission. Exclusion criteria were: history of hypotensive episodes, pre-existing or actual hypertensive or metabolic disorders, psychiatric or somatic disease, fetal/neonatal malformations, other contraindications for epidural analgesia. Informed consent was obtained from all individual participants included in the study.

## Epidural analgesia (EA)

After venous cannulation and survey of maternal parameters an epidural catheter was inserted at the L2-3 or L3-4 space. A bolus of 20 mL levobupivacaine and 10 μg of sufentanyl was subsequently administered, followed by a continuous infusion of a 10 mL/hour solution of either 0.0625% levobupivacaine with sufentanyl 0.5 μg/mL.

## Hemodynamic evaluation

Hemodynamic pattern was assessed using a non-invasive ultrasonic monitor (USCOM^®^, USCOM Ltd, NSW, Australia), used for the cardiovascular evaluation in pregnancy and validated versus echocardiography [15]. A transducer was placed on the suprasternal notch to measure transaortic or transpulmonary blood flow, respectively. At least three consecutive cycles were registered for each scan, by two trained researchers, to obtain the main cardiac parameters including total vascular resistances (TVR), heart rate (HR), stroke volume (SV), cardiac output (CO), arterial blood pressure. These measurements were obtained before (T0) and after 5 min 5 (T1) from epidural bolus, and at the end of the first stage of labor (T2).

## Computerized cardiotocography (cCTG)

The cCTG was performed for 1 h after epidural bolus by Sonicaid Oxford 8002 System (Manor Way, Old Woking, Surrey, England). Short-term variation (STV) was calculated as the average of sequential 1y16 minute pulse interval differences by Dawes-Redman software-based algorithm.

The protocol of this prospective study was approved by the ethics committee of our center and written informed consent was obtained from each patient.

## Statistical analysis

Comparisons were performed using Pearson chi-squared test for proportions, and using independent samples *t*-test or the Kruskal–Wallis test for continuous data. Descriptive data were analyzed using IBM SPSS Statistics for Windows, Version 22.0 (IBM Corp Armonk, NY, USA). A *P* value < 0.05 was considered statistically significant. This study was performed in line with the principles of the Declaration of Helsinki. This is an observational study. The internal academic Research Ethics Committee has confirmed that no ethical approval is required.

## Results

**Fig. 1:**
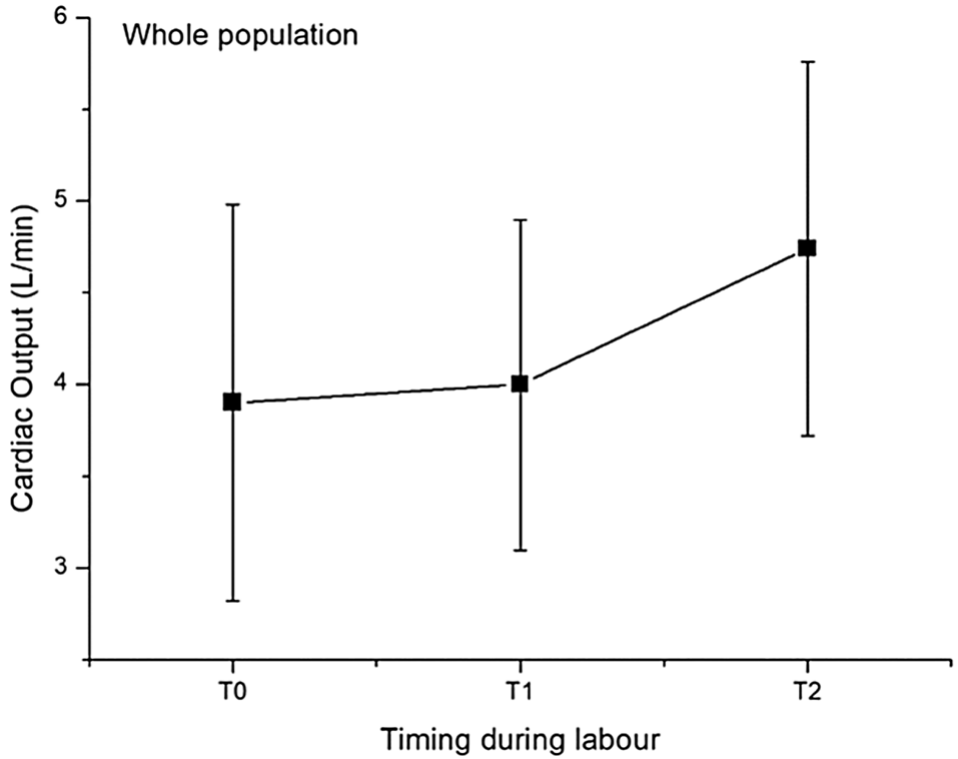
Cardiac Output during labor in the whole population studied

**Fig. 2:**
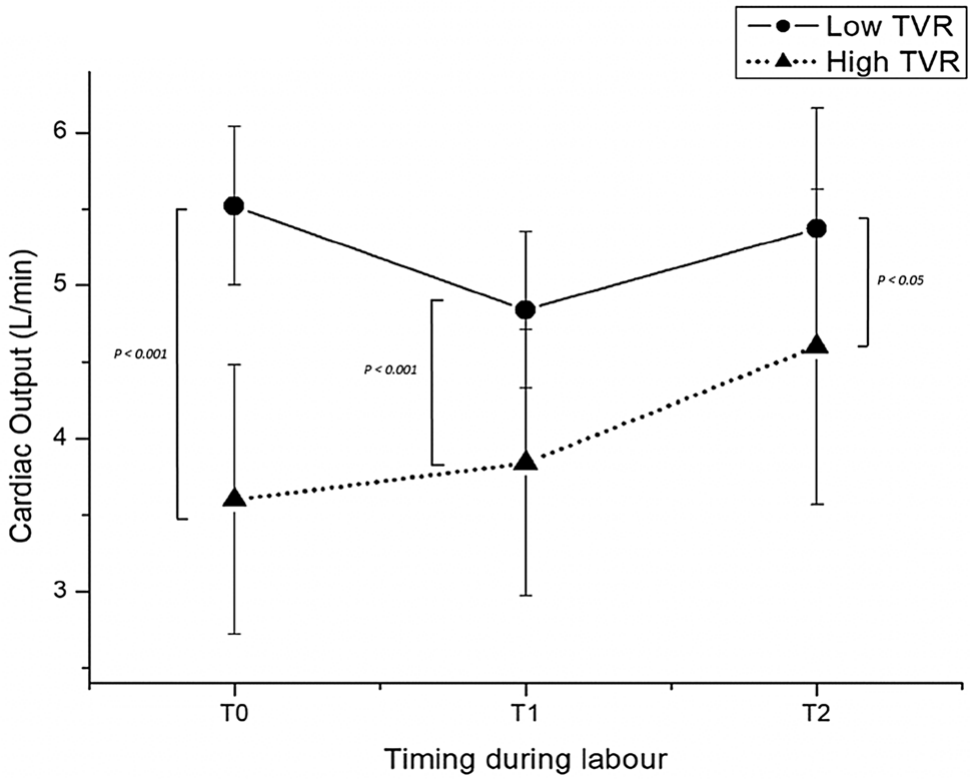
Cardiac Output during labor in the subgroup with Low Total Vascular Resistances (Low-TVR solid line) and in the subgroup with high Total Vascular Resistances (High-TVR dotted line)

**Fig. 3:**
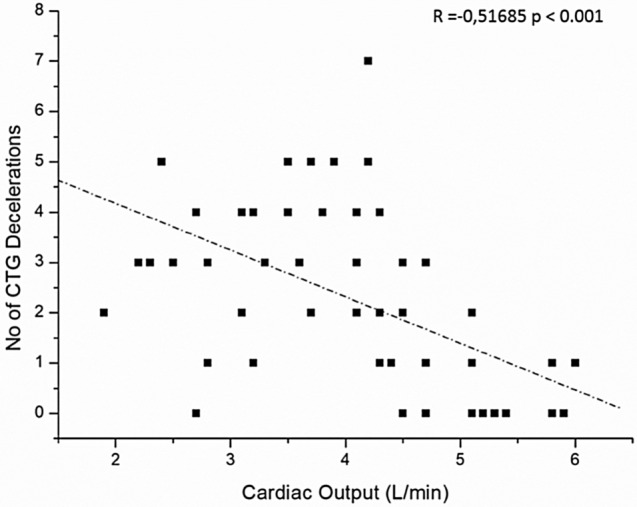
Number of decelerations at computerized cardiotocography related to Cardiac Output

**Fig. 4:**
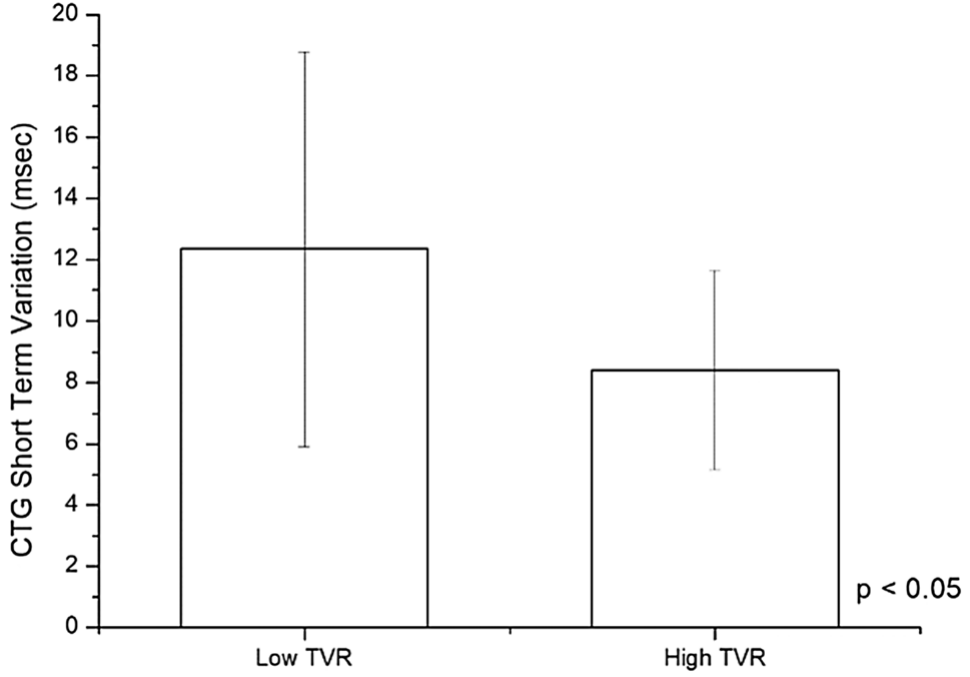
Short term variation at computerized cardiotocography in the subgroups with low Total Vascular Resistances (Low-TVR) and with high Total Vascular Resistances (High-TVR)

The 55 Patients recruited were divided in two subgroups Low-TVR and High-TVR using the reported cut-off 1200 dyne/sec/cm^−5^ [16, 17]. Characteristics of the study population are resumed in the Table 1. No significant differences were found in the characteristics of the two groups, not even in the rate of cesarean sections and in neonatal outcomes. Hemodynamics and cCTG records are summarized in the Tables 2 and 3. In the whole population Cardiac Output (CO) underwent a slight increase after epidural analgesia (EA) and a significant increase for the remainder of the first stage of labor (Fig. 1). Analyzing the CO trend by dividing the two subgroups we noticed that in the Low-TVR group the starting CO was significantly higher than in the High-TVR group (5.52 ± 0.52 vs 3.60 ± 0.88 L/min) (Fig. 2). After the bolus of epidural analgesia in the Low-TVR group there was a significant reduction in CO and then increased again at the end of the first stage, in the High-TVR group the CO increased insignificantly after the anesthesia bolus, while it increased significantly in the remaining part of the first stage of labor (Fig. 3). On the other hand, CO was inversely related with the number of decelerations detected on cCTG in the 1 hour after the epidural bolus (*R* = − 0.1685; *p* < 0.0001) (Fig. 4) while the Short-term variation was significantly lower in the group with High-TVR (Fig. 4).

**Table 1:** Characteristics of study population divided according to the level of the total vascular resistances (TVR) at admission (cut-off: 1200 dyne/sec/cm^−5^)

	Whole population	Low TVR	High TVR	*P*** < **
No. patients	55	39	16	
Age (years)	31.55 ± 5.36	30.20 ± 6.61	30.55 ± 5.91	n.s
BMI (Kg/m^2^)	24.13 ± 4.83	23.22 ± 2.74	23.53 ± 4.34	n.s
Gestational weeks	39.74 ± 0.91	40.2 ± 0.83	39.77 ± 0.86	n.s
Birthwheight (g)	3437.45 ± 343.26	3490.12 ± 339.31	3381.20 ± 398.15	n.s
Rate of cesarean section	18.1% [10]	8.5% [4]	15.3% [6]	n.s
Apgar Score 1’	8.86 ± 0.94	9.00 ± 0.35	8.60 ± 0.89	n.s

**Table 2:** Total vascular resistances

Low TVR Group	T0	*p* <	T1	*p* <	T2	*p *<
TVR (dyne/sec/cm^−5^)	1161.19 ± 178.27	< 0.05	1278.61 ± 271.13	n.s	1279.25 ± 393.25	n.s
CO (L/min)	5.52 ± 0.52	< 0.001	4.84 ± 0.51	< 0.05	5.37 ± 0.79	n.s
HR (bpm)	77.40 ± 9.31	n.s	74.20 ± 10.32	< 0.001	89.25 ± 11.34	< 0.001
SV (mL)	72.20 ± 9.54	< 0.001	65.60 ± 4.87	< 0.001	58.75 ± 8.18	< 0.001
SBP (mmHg)	108.21 ± 17.88	n.s	104.32 ± 13.41	< 0.05	111.25 ± 14.36	n.s
DBP (mmHg)	64.32 ± 8.94	n.s	61.57 ± 7.89	n.s	63.75 ± 14.93	n.s
cCTG STV (msec)			12.35 ± 6.45			
cCTG Decelerations			0.66 ± 2.33			

*CO* cardiac output, *HR* heart rate, *SV* stroke volume, *SBP* systolic blood pressure, *DBP* diastolic blood pressure, *cCTG STV* computerized CTG short term variation

**Table 3:** Total vascular resistances

High TVR Group	T0	*P* <	T1	*P* <	T2	*P* <
TVR (dyne/sec/cm^− 5^)	2093.96 ± 615.31	n.s.	1846.22 ± 481.52	n.s.	1668.44 ± 438.37	< 0.05
CO (L/min)	3.6 ± 0.88	n.s.	3.84 ± 0.87	< 0.05	4.61 ± 1.03	< 0.05
HR (bpm)	88.88 ± 11.78	n.s.	80.03 ± 16.11	n.s.	91.66 ± 18.10	n.s.
SV (mL)	42.70 ± 13.73	n.s.	49.44 ± 12.71	n.s.	51.94 ± 13.08	n.s.
SBP (mmHg)	116.48 ± 12.56	n.s.	111.66 ± 12.86	n.s.	119.16 ± 11.14	n.s.
DBP (mmHg)	71.48 ± 9.58	n.s.	68.70 ± 8.50	n.s.	71.11 ± 9.00	n.s.
cCTG STV (msec)			8.4 ± 3.23			
cCTG Decelerations			2.45 ± 5.25			

*CO* cardiac output, *HR* Heart rate, *SV* stroke volume, *SBP* systolic blood pressure, *DBP* diastolic blood pressure, *cCTG STV* computerized CTG short term variation

## Discussion

The correlated effects of epidural analgesia during labor have been extensively studied, nevertheless few studies have evaluated the phenomenon from the point of view of maternal hemodynamics. The main finding of this study was that if patients are selected on the basis of total vascular resistance, the hemodynamic attitude during labor and the response to epidural analgesia change significantly. We have shown that low vascular resistances are associated with higher levels of cardiac output and that this seems to guarantee better utero-placental and fetal performance during labor. Cardiac Output is calculated from stroke volume multiplied by heart rate, it increases throughout pregnancy as early as in the 5 week of pregnancy reaching in the three trimester, about 30–50% above that in the nonpregnant state [18]. Echocardiography is most commonly used for hemodynamics in pregnancy, invasive techniques are seldom used. An insufficient increasing of cardiac output during pregnancy has been associated to neonatal complications [19]. The influence of labor on hemodynamic values has been controversial, according to some authors there would be an increase in resting CO up to 50% [20–22] according to others there would be no changes [23]. According with previous evidences that CO may be linked to a fetal distress [16, 17], the hypothesis is that a lower CO, and thus a lower cardiac index, can affect fetal well-being as an expression of reduced cardiac performance and therefore of a reduced utero-placental perfusion.

Despite a physiological progressive reduction of vascular resistance by action of pregnancy mediators (nitric oxide, progesterone, prostaglandins,) and to the development of a low resistance circuit to the placenta, Doppler studies have associated the high resistance in uterine arteries to high peripheral vascular resistance and low maternal cardiac output [24].

The uterine fraction of maternal CO has been reported to be about 12% at term [25] on the other hand It has been calculated that utero/placental perfusion can be reduced by at least 60% during a uterine contraction in labor [26]. In the time of a contraction, most fetuses resist a period of short hypoxia, while fetuses with lower hypoxic tolerance limits show signs of compensation detected by the CTG in the form of decelerations and reduced variability. Short term variation (STV) is affirmed as a good predictor for fetal acid–base status during pregnancy and despite there are demonstrations of a significant increase in short and long term variation in peripartum period [27] in our series, in women with high-TVR, STV after an epidural bolus was significantly lower than in women with low-TVR, on the other hand women with low TVR and higher levels of CO have an improuved fetal response to maternal hypotension induced by epidural analgesic drugs as demonstrated by the reduced number of decelerations and higher short term variation.

Although with the limit of a relatively small sample size, the present study confirms a close link between maternal hemodynamics and uterus placental pathophysiology and studies the practice of epidural analgesia from a different point of view that allows to recognize differences substantial among women in labor. Childbear outcomes may be closely related to maternal low cardiac reserves, selecting a cohort of women in whom epidural analgesia can further worsen the hemodynamic stress of labor.
